# More than a silent Tsunami every year

**DOI:** 10.4103/0972-2327.40218

**Published:** 2008

**Authors:** Sanjeev V. Thomas

**Affiliations:** Professor of Neurology and Editor, Annals of Indian Academy of Neurology, Department of Neurology, Sree Chitra Tirunal Institute for Medical Sciences and Technology, Trivandrum - 695 011, India. E-mail: sanjeev@sctimst.ac.in

Let me wish all readers a Happy New Year. AIAN has entered the eleventh year of publication. The Academy and the journal have grown substantially in the past years. Nevertheless, there are major challenges that we have to face. In India, at least one person dies from rabies every twenty minutes. More than 25,000 deaths from rabies were reported from India to the WHO in the year 2004. This figure is almost twice the number of deaths during the tsunami that year. The annual incidence of rabies in India is estimated to be 2 per 100,000 population.[1] Data for subsequent years are not available. Unfortunately, all these are completely preventable deaths. A nation-wide WHO-sponsored survey in India[2] revealed that the annual incidence of animal bite was as high as 1.7%. It was more in the rural area, for children and lower socioeconomic group. Nearly, one-third of those bitten did not wash their wounds with soap or water, a preliminary, yet very important first aid. Nearly, half the victims used only nerve-tissue-derived vaccines, which are strongly discouraged by WHO because of its poor efficacy and adverse effects. Rabies immunoglobulin was used only rarely (2.6%). The high cost, poor availability of safe and efficacious vaccines and immunoglobulins, lack of awareness of the correct regimes for post exposure prophylaxis and complacency contribute to the high mortality of rabies in India. Current recommendations include intradermal injections of Purified Chick Embryo Vaccine or Purified Vero Cell Rabies vaccine with which adequate antibody levels are achieved in less than ten days time. There had been relatively less research into the pathogenesis or therapy of this fatal disorder. There were only 152 publications in 2007 in journals indexed with PubMed and only 8 publications from India, which has the highest mortality from rabies. This reflects the divergence of medical research from the pressing needs experienced in India. Strangely enough, the pharmacological treatment of rabies with antiviral drugs or other agents have not been addressed except for one or two publications. It is possible that human monoclonal antibodies may prove superior alternative to rabies immunoglobulin in the treatment of class III exposures. Stray dog bites account for most of the rabies exposures in India. Immunization of pet dogs had been a daunting challenge for this country, leave alone the immunization of stray dogs. Scientists in China have successfully developed an oral-bait-based vaccine for the immunization of stray dogs and other animals.[3] We need to have a more comprehensive scientific approach to the entire problem of rabies in this country. The epidemiological aspects, basic science component, clinical aspects, treatment and prophylaxis need intensive research in this country. In this issue of AIAN, we have included a comprehensive review on antemortem diagnosis and management of rabies.

The quality of life (QOL) evaluation is gaining more importance in clinical practice and medical research. This is an attempt to estimate the quality of life from a patient's perspective rather than from a health care providers viewpoint. There are several generic instruments that assess health-related quality of life, such as SF 36 and WHOQOL. There are other disease specific instruments like QOLIE and FACIT. Some instruments are specially designed for children or adolescent subjects. The QOL is closely linked to the culture, heritage and value systems of a given geographic area. Hence, it is important that the instrument that one uses is appropriately designed and content validated. Whenever an internationally popular instrument is used, it is important to ensure that the translation had been accurate and the new instrument is well validated. In this issue of AIAN, we have two articles that deal with HRQOL. One of them is related to epilepsy and the other is related to Wilson's disease. I hope the readers will find these articles useful.

Most clinicians practicing in India are not familiar with the term advocacy, although many of them had been contributing to a lesser or greater extent to this concept. Advocacy refers to the act of pleading or arguing in favor of a cause, idea or policy. The important objectives of advocacy for neurologists are promotion of human rights of the persons with neurological disorders and of their families and monitoring the life conditions of people with these disorders and their families. We would have to campaign with decision makers, interact with media, empower persons with neurological disorders and their support groups and fight against the stigma attached to some of the neurological disorders such as epilepsy. The American Academy of Neurology had been conducting a series of workshops to train neurologists in advocacy. In a country such as India, neurologists have an important role to play in advocacy. I would like to draw the reader's attention to this viewpoint through this issue.
